# An optical imaging chamber for viewing living plant cells and tissues at high resolution for extended periods

**DOI:** 10.1186/s13007-015-0065-7

**Published:** 2015-03-22

**Authors:** Grant Calder, Chris Hindle, Jordi Chan, Peter Shaw

**Affiliations:** Department of Cell & Developmental Biology, John Innes Centre, Norwich Research Park, Norwich, NR4 7UH UK

**Keywords:** Arabidopsis, Imaging chamber, Time-lapse microscopy, Perfusion, Live cell imaging, Plant tissue

## Abstract

**Background:**

Recent developments in both microscopy and fluorescent protein technologies have made live imaging a powerful tool for the study of plant cells. However, the complications of keeping plant material alive during a long duration experiment while maintaining maximum resolution has limited the use of these methods.

**Results:**

Here, we describe an imaging chamber designed to overcome these limitations, which is flexible enough to support a range of sizes of plant materials. We were able use confocal microscopy to follow growth and development of plant cells and tissues over several days. The chamber design is based on a perfusion system, so that the addition of drugs and other experimental treatments are also possible.

**Conclusions:**

In this article we present a design of imaging chamber that makes it possible to image plant material with high resolution for extended periods of time.

**Electronic supplementary material:**

The online version of this article (doi:10.1186/s13007-015-0065-7) contains supplementary material, which is available to authorized users.

## Background

The progress in optical microscopy technology (faster acquisition, more sensitive cameras, specialised immersion objectives, confocal, multi photon etc.) combined with direct labelling of target proteins using fluorescent protein markers has made live cell imaging a powerful tool for investigating dynamic processes in plants [1-5] This technology enables us to follow dynamic events in live experiments, rather than taking snapshot images of chemically fixed material. This allows many biological processes to be studied from start to finish. For example, the processes involved in the formation of a tissue or organ are regulated by developmental cues or circadian rhythms. To understand these developmentally regulated patterns it is necessary to follow cells in a developing tissue throughout organ formation, which can take from hours to days [6].

Short term imaging of living samples is relatively easy and the sample preparation can be as simple as putting the sample onto a slide in a suitable medium with a cover glass on top. However, successful high resolution imaging for a long period of time is more difficult. Three key factors need to be addressed: a constant environment surrounding the sample; stably holding the sample’s position within the imaging volume; and, for larger samples, imaging a large enough volume. To track a sample for long durations it is necessary to make specialised sample holders or chambers to address these issues [7].

In general plant material is very robust to large variations in many environmental conditions. However, plant samples are highly susceptible to decreasing levels of nutrients and oxygen (anoxia) during live cell imaging experiments. Effects like anoxia have been shown to alter the localisation of a range nuclear and nucleolar proteins from a nucleoplasmic distribution to a speckled pattern [8]. Therefore imaging chambers for plants must be designed to maintain the sample’s environment, in order to keep the sample healthy over periods of days. In static systems, as a plant grows, the nutrients in the medium are used up, changing the specimen’s environment. It is therefore preferable to use a perfusion system to replace depleted media (nutrients and oxygen) around the sample, giving a stable environment throughout the duration of the experiment.

When imaging at high resolution, optical constraints limit the imaging volume. A typical high magnification immersion objective has a working distance in z (the optical axis) of 100–200 μm and a field of view in x and y (the image plane) of 200-400 μm. It is therefore necessary to position different sized samples within this working distance (close to the cover-glass) without damaging them, and then stably hold their position within this narrow imaging volume throughout an experiment. An additional stability issue can be caused by the laminar flow of fresh media into the chamber when using a perfusion-based system.

The microscope’s field of view is a limiting factor in imaging large or fast-growing specimens. Low magnification lenses can be used to increase the viewable area but this decreases the resolution. To collect high resolution images of large specimens, it is necessary to increase the field of view of the microscope by another means. This can be achieved by translating the specimen to create a series of overlapping images, which can be stitched together to form a high resolution tiled image. This technique requires a computer-controlled motorised x-y stage and suitable image processing software to stitch the images together such as, for example, ImageJ/Fiji [9-12]. Most microscope manufactures are now able to equip their systems with motorised stages and image stitching software.

Although there are several good commercial imaging chambers available [13], these chambers have not been specifically designed for the high resolution imaging of plant tissues over extended periods. Instead, commercial systems are primarily designed for imaging animal and microbial cell cultures using inverted microscopes. However, gravitropism in plants causes some complications, since aerial parts such as leaves tend to grow upwards and roots grow downwards. Since it damages the plant to clamp it too tightly, this generally means that the smaller parts – usually those parts of interest that are growing rapidly – are not fully constrained between the front and back of the chamber. Thus, leaves will tend to grow away from the coverglass on an inverted system, but will stay next to it on an upright system. Conversely, roots will tend grow away from the coverglass on an upright system, but stay on the coverglass on an inverted system. This means that it is preferable to have a growth chamber that can be accommodated on either an upright or an inverted microscope. Other things being equal, we prefer the ease of use of an upright microscope.

In plants the majority of long duration time-lapse experiments have either been performed using static systems, which cause the environment issues discussed above over extended periods [7], or using perfusion systems designed specifically for the study of roots on inverted microscopes [14].

Our aim, therefore, was to construct an imaging chamber that allowed the stable imaging of a range of plant tissues over extended periods of time at high resolution. Here we describe the design of a fully enclosed imaging chamber based on a liquid perfusion system that can be attached to an upright microscope, or could be easily used in an inverted orientation adapted for an inverted microscope. This modified perfusion chamber is designed to reduce the negative effects of laminar flow of medium. It has the additional benefit that the environmental medium can be changed or drugs or other soluble components can be added during an imaging experiment, which makes this system a powerful experimental tool.

## Results and discussion

### The imaging chamber

**Figure 1:**
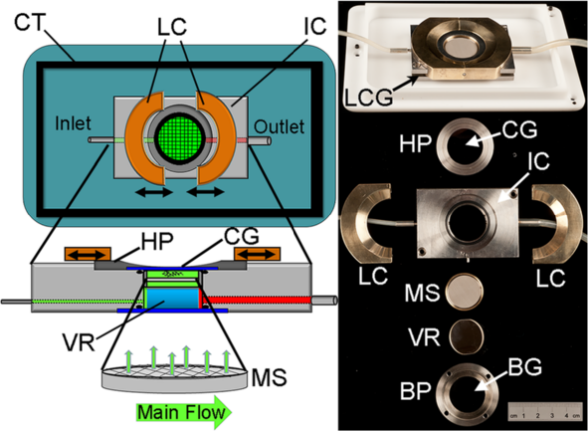
**Design of the imaging chamber.** The left panel is a schematic diagram of the chamber from top and side views. Note that the flexible mesh support removes the sample from the laminar flow of the incoming liquid protecting it from shear stress. The right panel is a picture of the chamber fully assembled and disassembled. CT - Containment tray, HP - holding plate, IC - imaging chamber, LC - locking clamp, MS - mesh support, VR - volume reducer, BP - base plate, CG - cover glass, BG - base glass, LCG – locking clamp groove.

The system is designed in two parts: an outer containment tray (CT in Figure 1 – L160mm x W110mm x H16mm) that attaches to the microscope stage; and an inner specimen chamber (IC in Figure 1 – L70mm x W48mm x H11mm). The outer tray was designed to fit into a Prior Proscan H101 motorised scanning stage, but is easily attached to different brands of microscope stages with simple adapters. The outer containment tray protects the microscope from possible damage from the liquid medium if the imaging chamber should leak. The imaging chamber is made from stainless steel and glass, which can be sterilised by autoclaving prior to use to minimise any potential contamination problems. An optical cover glass (CG in Figure 1) with a 32 mm diameter and a thickness of 0.17 mm (number 1.5 - Thermo Scientific) is used; this thickness is necessary for high resolution immersion objectives. As the cover glass is very thin it requires a metal holding plate to give added structural strength and rigidity (HP in Figure 1 - outer diameter 40 mm, inner diameter 25 mm, thickness 1 mm). The holding plate has a 25 mm opening but only 20 mm area is accessible due to the physical constraints of immersion objective lenses. The cover glass and holding plate are held in place on the imaging chamber by two sliding locking clamps (LC in Figure 1 - each one L35mm x W55mm x H10mm, with a 35 mm diameter semi-circular cut-out for HP) that have flanges (6 mm × 3 mm) that slide in slots in the inner chamber (LCG in Figure 1). When the two clamps are pushed together the holding plate and coverglass are lightly but firmly held in position by an even force that minimises physical distortion. With this sliding clamp configuration the imaging chamber can be easily opened and resealed to allow the quick addition of biological material, usually under sterile flow hood conditions. To open the chamber, perfusion is stopped and the outlet tube is clamped closed to prevent medium from draining from the chamber. The locking clamps are slid apart along the chamber body to expose the holding plate, which can then be removed to give access to the sample. The chamber is reassembled in the reverse order. The base of the imaging chamber is made in two parts - a base glass (BG in Figure 1 - window 35 mm diameter, thickness 2 mm - UQG Optics Ltd), which is held in place with a base plate (BP in Figure 1 - outer diameter 44 mm, inner diameter 25 mm, thickness 2.5 mm), screwed into the body of imaging chamber. The imaging chamber body contains top and bottom O-ring gaskets to create watertight seals between both the cover and base glasses. To lower the cost of an experiment using expensive compounds, the imaging chamber volume can be reduced from 3.5 ml to 2.0 ml by inserting a circular glass volume reducer (VR in Figure 1 - 25 mm diameter, thickness 3 mm) (UQG Optics Ltd).

The specimen is placed on top of a thin stainless steel mesh (Endecotts Ltd) stretched out across a supporting ring to form a mesh support (MS in Figure 1 - 26 mm diameter, thickness 3 mm). The height of the mesh support is adjustable and is usually set to approximately 100-150 μm from the cover glass to form a suitable cavity for Arabidopsis seedlings. The mesh support positions the sample near the cover glass so that it is within the working distance of an immersion objective lens. The mesh is flexible and deforms to the shape of the tissue to some extent, which helps to stabilise the specimen’s position in z (the direction of the optical axis). Previous designs of perfusion imaging chambers suffered from sample movement or damage caused by the laminar flow of medium passing through the narrow sample cavity. This chamber design alleviates this problem by using the mesh support to separate the sample cavity from the flowing medium. Medium is exchanged between the two sides by passive diffusion of liquid through the 20 μm pores in the mesh (Figure 1).

A peristaltic pump is used for long duration experiments with large media reservoirs. For adding soluble compounds a syringe pump is more convenient as syringes containing different compounds can be quickly exchanged and the solution can be added manually, allowing for faster application. The flow rate for the peristaltic pump was measured directly. For the syringe pump a calibration table relating the size of syringe and the pump speed was used. The input pressure drives the flow through the chamber, and there is no output pump. The culture medium is pumped into the imaging chamber through an inlet tube of 1 mm inner diameter and exits through a larger 2 mm inner diameter outlet tube, which prevents pressure build-up inside the chamber.

### Environmental conditions

**Figure 2:**
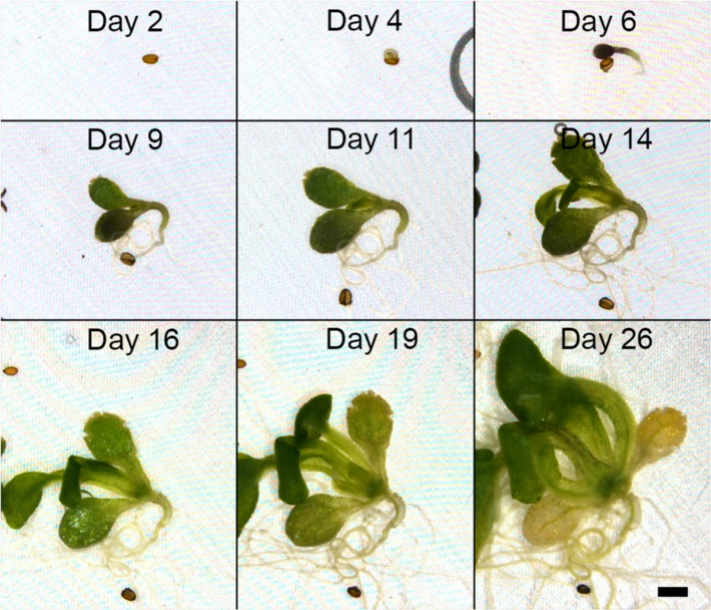
**Arabidopsis seedling growth inside imaging chamber.** Low magnification images showing the germination and growth of an Arabidopsis plant inside the imaging chamber. The moiré patterns in the background are caused by interference between the illumination and the grid of the mesh support. Scale bar = 1 mm.

A successful imaging chamber needs to maintain a healthy environment over extended periods. This system continuously perfuses fresh media into the chamber to replace depleted nutrients. To assess the system’s ability to support plant growth we placed Arabidopsis seeds inside the chamber and then imaged them at daily intervals using a stereo microscope. This experiment showed that seeds could germinate inside the chamber and that the perfusion system provided adequate nutrients for growth into large seedlings, which developed root and leaf systems. An example of germination is shown in Figure 2 (also see movie Additional file 1). The seed germinated after 4 days, the hypocotyl emerging from the seed coat. This was then followed by the expansion of the hypocotyl and cotyledons. New leaves and roots were then seen to emerge from day 9 and continued to grow throughout the duration of the experiment (31 days). These results demonstrate that the system is suitable for tracking the expansion of tissues such as the hypocotyl [15] and cotyledons as well as to follow development of emerging roots and leaves [16].

Here, we imaged for 31 days to demonstrate the system’s ability to maintain growth. The growing plant was continuously perfused over the entire period using a peristaltic pump and a 1 litre reservoir of medium which was continuously recycled (the total amount of nutrients used was very small compared to the size of the reservoir, and oxygen was replenished by atmospheric exchange during recycling as the reservoir was not airtight). However, it is important to note that over these extended periods (tens of days) the depth of chamber’s sample cavity set at the start of the experiment imposes a physical constraint that can cause tissue squashing or distortion during long periods of growth. To prevent extreme changes in specimen size during experiments, we recommend using specimens at the appropriate developmental stage and adjusting the chamber’s internal cavity depth using the mesh support (MS in Figure 1) to match the specimen’s size.

### Maintaining sample position

**Figure 3:**
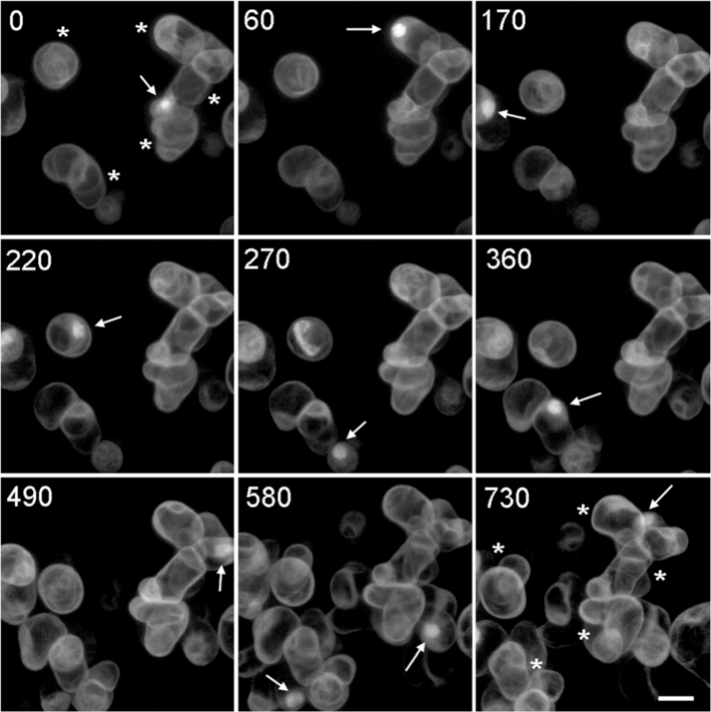
**Time-lapse of Arabidopsis suspension cells.** Confocal imaging of Arabidopsis suspension cells expressing GFP tubulin. A large field of view containing several cells was imaged every 10 minutes for 780 minutes. Cell growth and division (arrows) were captured throughout experiment with asterisks indicating examples of cells that have maintained stable positions. Scale bar = 20 μm.

Time-lapse experiments using high resolution immersion objective lenses commonly suffer from the part of the specimen of interest drifting out of the small and narrow imaging volume. It is therefore imperative that the imaging chamber stably holds the specimen’s position. With a perfusion system used to maintain the sample’s health, the flow of fresh medium flowing into the chamber could cause a problem by affecting the sample’s position. To prevent this we used a design that separates the sample from the direct flow of medium with a mesh support (MS in Figure 1). We used suspension cells to test the chambers ability to maintain a sample’s position, since they exist in small loosely packed groups which are more liable to move out of the imaging volume if strong currents are generated by media flow. In this experiment, Arabidopsis culture cells expressing GFP-tubulin were added to the chamber and allowed to settle for a period of 2 hours. Confocal section stacks were collected every 10 minutes for 14 hours (Figure 3). Despite nearly continuous imaging, the cells underwent growth and division (see white arrows) indicating that they were healthy. Some movement was caused by cell growth but most cells remained within the imaging volume (marked by asterisks in Figure 3, first and last time-points) showing that the flow of fresh medium did not seriously affect the specimen position.

### High resolution imaging

**Figure 4:**
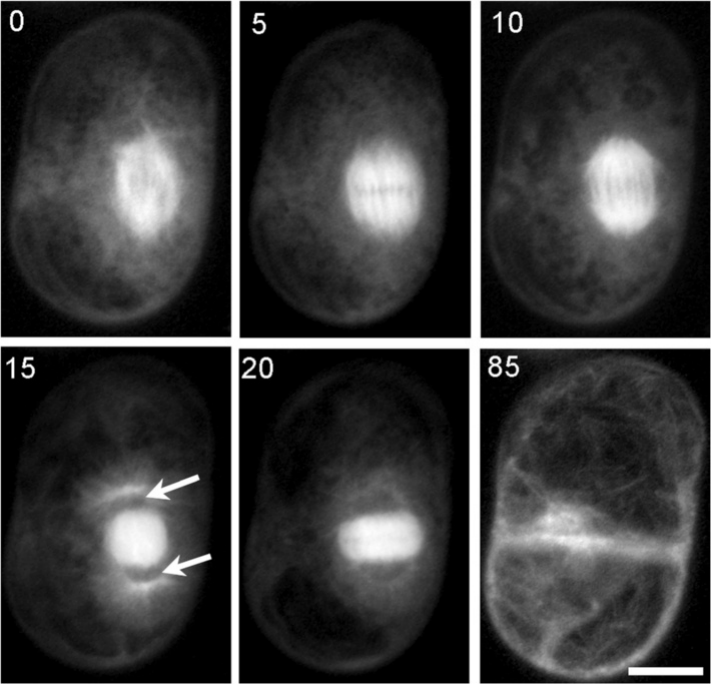
**High resolution imaging of a dividing Arabidopsis suspension cell.** Confocal microscopy time-lapse images of an Arabidopsis culture cell expressing GFP tubulin. High resolution z stack images were collected using a 63/1.4 oil immersion objective, every 5 minutes throughout division. This illustrates the interchange of different microtubule structures: prometaphase (0 min); metaphase (5 min); anaphase (10 min); telophase (15 min daughter nuclei position indicated by arrows); phragmoplast (20 min); cell division completion (85 min). The numbers indicate the time in minutes from start of time-lapse acquisition and confocal z stacks are shown as summation projections. Scale bar = 10 μm.

Detailed images of sub-cellular structures such as microtubules are only possible if a high resolution immersion objective is used. In turn this will limit how far from the cover glass the sample can be positioned - typically a maximum of about 200 μm. The chamber’s design allows the sample to be positioned within this small working distance. Figure 4 shows Arabidopsis suspension cells expressing GFP-tubulin imaged using a confocal microscope equipped with 63/1.4 oil objective lens, which has a working distance of 190 μm. The chamber’s mesh support was set to a depth of 100 μm to position the cells within the working distance of the objective. During this experiment, confocal section stacks were collected every 5 minutes for 4 hours. Detailed images of a cell as it progressed through cell division revealed microtubule structures from pro-metaphase through to cytokinesis (see Figure 4 and Additional file 2). This shows that the chamber supports high resolution optics, allowing detailed imaging of subcellular structures.

### Imaging large samples

**Figure 5:**
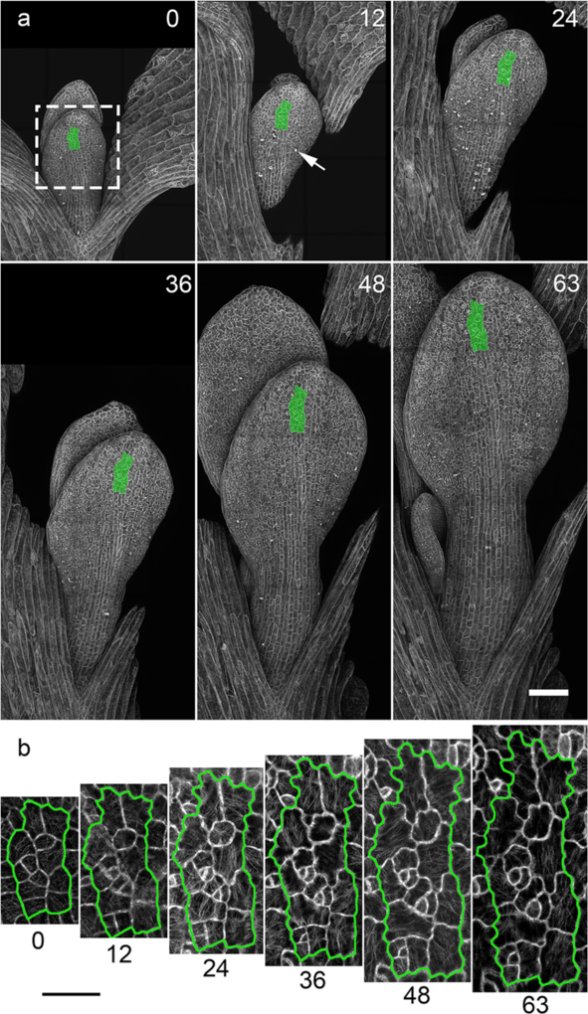
**Increasing the field of view using tiled imaging to capture the growth of an Arabidopsis leaf expressing GFP tubulin. (a)** The dashed box indicates the limited field of view from 40/1.2 water objective at a single position. Tiled images were captured every hour for 63 hours where cell division (arrow) and growth were captured. Scale bar = 100 μm. **(b)** Displays a sub region from the tiled image (panel (a) highlighted in green) illustrating cellular growth. Maximum projections of the z stacks are shown and the numbers indicate time in hours from start of time-lapse experiment. Scale bar = 30 μm.

With a high resolution objective the available field of view is limited and plants can quickly grow beyond this area. The chamber was designed to attach to a motorised stage so that image tiling can be used to increase the area imaged. The stage moves the sample to create a series of overlapping images, which are stitched together to form a larger image. To illustrate how image tiling can be used to track the growth of plant tissue at high resolution, we used a confocal microscope equipped with a motorised stage and a 40x/1.2NA water immersion objective to follow the growth of an Arabidopsis leaf expressing GFP-tubulin. Overlapping confocal section stacks of the growing leaf were collected every hour for 63 hours (Figure 5 and Additional file 3). The available imaging area of this objective lens was limited to 212 × 212 μm (see the dashed box in Figure 5a). At the start of the experiment a 3 × 3 array of overlapping images (595 × 595 μm) was used to collect an image of a complete leaf. By the end of the experiment, the area needed to image the entire leaf had increased, to a 3 ×7 array of overlapping images (1360 × 595 μm). It was possible to follow cell growth and division (arrow at 12 hours shows a mitotic cell) as the leaf developed. Tracking a complete developing organ can reveal how cell division and growth are regulated at the tissue level, for example in hypocotyls [15] and leaf tissue [6,16]. To illustrate cell growth we have extracted from the tile images a smaller sub region of cells near the leaf tip (highlighted in green in Figure 5a) and displayed them in Figure 5b. This sub-region of cells shows the high resolution information that is available with the tiling approach. Some caution is needed when overlapping a large number of areas since, if significant growth occurs during the long acquisition time necessary, the final tiled image will be distorted.

### Changing the specimen environment

**Figure 6:**
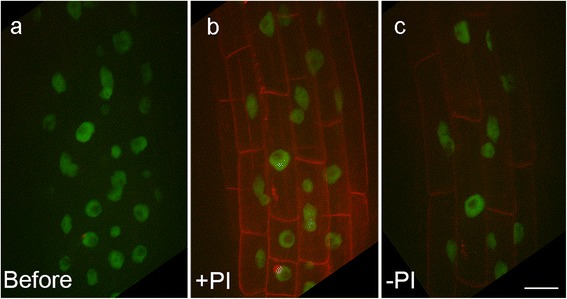
**The addition and removal of PI in a growing Arabidopsis root during image acquisition.** The root was imaged by confocal microscopy where z stacks were taken every 5 minutes. **(a)** Only green fluorescent nuclei (GFP-eIF4A-III) are visible at the start of the experiment **(b)** Within 5 minutes after the addition of PI cell outlines become clearly visible **(c)** PI was washed out of the chamber leaving only a faint red outline. Images are summation projection of the z stacks. Scale bar = 20 μm.

An advantage of a perfusion system over a static one [7] is that soluble compounds can be quickly added or removed from the imaging chamber and their effects can be tracked without disturbing the specimen. To illustrate this we added and removed propidium iodide (PI) from the chamber containing a growing Arabidopsis seedling expressing eIF4a-III-GFP, which is localised to the nucleus. Propidium iodide fluoresces in the red spectral region, and is excluded from the interior of living plant cells. It is commonly used to label the cell outlines in roots [17]. We used appearance and disappearance of PI fluorescence to monitor the addition and removal of the compound from the plant. Confocal section stacks of growing roots were collected every 5 minutes throughout the experiment (Figure 6 and Additional file 4). At the start of the experiment, before the addition of PI, only the root cell nuclei were visible (green in Figure 6a). PI was added to the medium by manually injecting 6mls of PI solution over 60 secs and then perfusing the chamber. Cell outlines became visible within 5 minutes (red in Figure 6b). To remove the PI, several chamber volumes (10 ml) of fresh MS media were then manually flushed through the system between two time-points 5 minutes apart, which removed most of the PI, leaving only very faint cell outlines from the small amount of residual PI (Figure 6c). It should be noted that although compounds can be added or removed from the chamber within minutes, the uptake or release rate from the specimen is tissue specific (for example, very fast in roots, but slower in leaf because of the waxy cuticle). The chamber’s design allows for uninterrupted imaging during the addition or removal compounds because the specimen is separated from the direct flow of incoming media. The ability to add or remove biologically active compounds makes this system a powerful experimental tool. In a recent example using this chamber gibberellic acid was added to growing Arabidopsis seedlings [15], and the hypocotyls were imaged.

## Conclusions

Long duration time-lapse imaging experiments of living plants at sub-cellular resolution present substantial challenges which require a specialised imaging chamber. Here we describe a suitable imaging chamber that can maintain plant material in a healthy state for extended period of time by replacing depleted nutrients with fresh media. Its flexible design enables imaging of a wide variety of plant specimens – cell cultures, hypocotyls, leaves and roots. The system is compatible with high resolution immersion objectives, allowing the samples to be positioned close enough to the cover glass to be within the working distance of high numerical aperture objective lenses. The mesh support helps to maintain the sample’s position within the imaging volume but is flexible enough to allow sample growth. The chamber can be attached to microscopes equipped with motorised x,y stages to allow larger samples to be imaged at high resolution by tiling overlapping images. This design of imaging chamber eliminates movement problems caused by the flow of incoming media that can be a problem in perfusion systems. The ability to add or remove soluble biologically active compounds without affecting image acquisition makes this imaging chamber a powerful experiment tool for studying plant development, which has already been used in many published studies.

## Methods

### Cell culture

Stable cell lines expressing an alpha tubulin 6-GFP fusion protein expressed under the 35S promoter were derived from *Arabidopsis thaliana* Col-0 root explants. Calli were then transferred to Murashige and Skoog liquid culture (MS: Formedium, Hunstanton, UK) containing 3% (w/v) sucrose (Sigma) and grown in the dark at 25°C with 150 rpm agitation and were sub-cultured every 7 to 8 days at a 1/10 dilution as previously described [18]. Cells were transferred to the microscope imaging chamber 2 days after sub-culturing, in a laminar flow hood to prevent contamination. After transfer to the imaging chamber, cells were continuously perfused with MS medium containing 1% (w/v) sucrose at a flow rate of 3 ml/h.

### Plant material

*Arabidopsis thaliana* plants expressing 35S::GFP-eIF4A-III [8] or 35S::alpha tubulin 6-GFP [19] were used. Seeds were sterilised in 5% (w/v) sodium hypochlorite before transfer onto Petri dishes containing MS medium containing 1% (w/v) sucrose (Sigma) and 0.5% (w/v) Phytagel (Sigma). Seeds were stratified at 4°C for 2 days and grown for 5 days for root experiments or 7 days for young leaf experiments under artificial light at 23°C with a 16 hour photoperiod. 5 or 7-day-old seedlings were transferred to the microscope imaging chamber in a laminar flow hood to prevent contamination and then imaged using a confocal microscope. After transfer the seedlings were continuously perfused with ¼ strength MS liquid medium at a flow rate of 3 ml/h.

### Low magnification imaging

Arabidopsis seeds were sterilised and placed inside the imaging chamber and continuously perfused with ¼ strength MS liquid medium at a flow rate of 3 ml/h. The chamber was housed in growth room under artificial light at 23°C with a 16 hour photoperiod. The chamber was removed briefly for low magnification imaging using a Leica M205 FA stereo microscope with both transmitted and reflected illumination. Images were collected over a period of 28 days at intervals of ≥1 day.

### Confocal imaging

Arabidopsis cell cultures were imaged with a Zeiss LSM 510 Meta confocal microscope using a 63/1.4 oil immersion objective lens. GFP fluorescence was excited using the 488 nm line from an argon ion laser and the emitted light was filtered through a 505-550 nm band-pass filter. Confocal z section stacks were collected at 1 μm spacing through the depth of the cultured cell, and time-series images were acquired with an interval of either 5 or 10 minutes.

Arabidopsis seedlings were imaged using a Zeiss LSM 780 confocal microscope using a 40/1.2 water immersion objective lens. GFP fluorescence was excited using the 488 nm line from an argon ion laser and the emitted light was capture using a GAsP spectral array 499-579 nm. Confocal z section stacks were collected at 2 μm spacing through the depth of the leaf tissue, and time-series images were acquired with an interval of 1 hour. Image tiling was controlled through Zeiss Zen Black software with 10% overlap between tiles for software alignment and stitching.

The Arabidopsis roots were imaged using a VisiTech QLC100 (Visitech, Sunderland, UK) spinning disc confocal microscope using a 40/1.3 NA oil objective lens. GFP and propidium iodide were both excited using the 488 nm line of a diode laser and emitted light was filtered through band-pass filters (500–550 nm for GFP and 580-654 nm for propidium iodide) and detected using a Hamamatsu Orca ER cooled CCD camera. Confocal z section stacks were collected at a spacing of 1.5 μm through the depth of the root tip and time-series images were acquired with an interval of 5 minutes.

### Adding soluble compounds

The red fluorescent dye propidium iodide was chosen to add to Arabidopsis roots expressing 35S::GFP-eIF4A-III as it concentrates in the apoplastic spaces between cells revealing the cell outlines [17]. The propidium iodide was added to the chamber by manually injecting 6 ml (i.e. 3x chamber volume exchanges as the chamber volume was 2 ml) of propidium iodide (10 μg/ml) dissolved in ¼ strength MS medium over approximately 60 secs (i.e. a rate of 360 ml/hour) and was further perfused for 1 hour at 3 ml/h. To remove the propidium iodide 10 ml of ¼ strength MS media was flushed manually through the chamber between two consecutive timepoints (5 minute intervals) and was further perfused at a rate of 3 ml/h.

### Image analysis

All images collected by confocal microscopy were processed using Fiji [9,12], a version of ImageJ [10]. Time-lapse movies of cell division and root growth were aligned using the StackReg plug-in [20] and the time-lapse movies were aligned manually using “AlignmentMacros”. Fiji was used to project z data using either maximum or sum projection. Fiji was also used to rotate, crop, adjust the brightness and contrast, and add scale bars and time stamp to movies [21]. The Zeiss Zen Black software was used to stitch overlapping images (10%) to form a larger tilled image. Adobe Photoshop was used for figure assembly and annotation.

## Additional files

Additional file 1:
**Arabidopsis plant growth inside the imaging chamber at low magnification (QuickTime movie).** A low magnification overview of developing Arabidopsis plants growing from seed to seedling as seen in Figure 2. Time interval ≥ 1 day. Scale bar = 1 mm. Additional file 2:
**Time-lapse of an Arabidopsis suspension cell going through cell division (QuickTime movie).** High resolution confocal microscopy imaging of a single Arabidopsis culture cell expressing GFP tubulin as it progresses through cell division as seen in Figure 3. Time interval = 5 minutes. Scale bar = 10 μm. Additional file 3:
**Tiled image time-lapse of a growing Arabidopsis leaf (QuickTime movie).** Time-lapse of a developing Arabidopsis leaf expressing tubulin GFP complete tissue captured using image tiling as seen in Figure 4 Time interval = 1 hour. Scale bar = 100 μm. Additional file 4:
**The addition and removal of PI from the chamber containing a growing Arabidopsis root (QuickTime movie).** The addition and removal of propidium iodide dye to an Arabidopsis root expressing GFP nucleus marker as seen in Figure 5. Time interval = 5 minutes. Scale bar = 20 μm.

## Abbreviations

MS: Murashige and Skoog
PI: Propidium Iodide
